# Controlling minor outbreaks is necessary to prepare for major pandemics

**DOI:** 10.1371/journal.pbio.3002945

**Published:** 2024-12-03

**Authors:** Adam J. Kucharski

**Affiliations:** Centre for Epidemic Preparedness and Response, London School of Hygiene & Tropical Medicine, London, United Kingdom

## Abstract

Ongoing influenza H5N1 outbreaks highlight the need for timely, scalable interventions that draw on lessons from COVID-19. This Perspective discussed that successful pandemic preparedness requires early outbreak management, including effective responses targeting spillovers before evidence of human-to-human transmission exists.

## Teaser

Ongoing influenza H5N1 outbreaks highlight the need for timely, scalable interventions that draw on lessons from COVID-19. In particular, successful pandemic preparedness requires early outbreak management, including effective responses targeting spillovers before there is evidence of human-to-human transmission.

Preparing for pandemics requires preparing for small outbreaks. Take avian influenza. As of 1 October 2024, there were 15 reported cases of H5 influenza in the United States (with 10 confirmed as H5N1), including a cluster of suspected cases without an animal source. Although most of these individuals would later test negative for H5N1 in antibody tests, in real-time—without the benefit of hindsight—such patterns may be indicative of human-to-human transmission [1]. If genuine H5N1 clusters do occur in future, it would not be the first time there has been spread of H5N1 among humans. During outbreaks in Indonesia during 2005 to 2009, there were several instances of household transmission clusters, with the reproduction number (i.e., the average number of people that a typical case spreads the infection to) estimated in the 0.1 to 0.25 range [2].

Fortunately, the ongoing spread of H5N1 is not a sudden novel threat. Unfortunately, the fragmented, stifled response to H5N1 in the US suggests that many are putting the threat firmly in an “isolated spillover” category, rather than viewing it more accurately as a pathogen with pandemic potential. Worse, the risk is not limited to the US. There have been outbreaks of H5N1 among sea lions and elephant seals in Argentina, with phylogenetic analysis suggesting both mammal-to-mammal and mammal-to-bird transmission [3]. This may be the first time in history we are witnessing the gradual emergence of a new pandemic virus in real-time. Or, of course, it may not be. The problem is that we cannot base our response on a future we do not yet know. In that sense, the term “pandemic preparedness” is something of a misnomer, because a pandemic never starts with a known outcome. It is only in hindsight that we can distinguish between isolated spillover events, localised outbreaks and full-blown pandemics. In the initial stages, all these events will look similar. For example, in early January 2020, epidemiological reports of a novel coronavirus were mostly consistent with a large zoonotic spillover event in Wuhan, rather than efficient human-to-human transmission [4]. It is therefore a major concern that the more opportunities H5N1 has to spread among mammals, the larger the probability it will adapt to human transmission [5].

Suppose more human H5N1 cases crop up in the US, alongside the many that are probably being undetected currently based on early serological data [6]. Then, suppose more transmission clusters start appearing, and more cases that haven’t had any animal contact. And finally, suppose we start seeing human cases exported to other countries, and evidence of high severity in certain age groups, alongside growing evidence of human-to-human transmission. How should countries respond in this situation? And how should they respond to other potentially pandemic pathogens?

One of the most effective points at which to intervene against an emerging disease threat is the point at which it spills over from animals to humans. When avian influenza H7N9 caused over 1,500 human infections in China during 2013 to 2018, efforts focused on reducing risk from the poultry reservoir via market closures and vaccination [7]. In many ways, this was the epitome of a “One Health” policy: tackling a predominantly animal and environmental health problem before it became a major human one.

Acting before human spillover first occurs is much harder. Although there have been attempts to predict pandemic pathogens before they emerge, new outbreaks are rare compared to the number of potential pathogens in animal reservoirs, limiting our ability to validate predictions [8]. New pathogen variants may also emerge in the future, reducing the relevance of any previous reservoir sampling efforts.

Intervening at the later stages of the emergence process is also challenging. If countries wait until human-to-human transmission has established, it creates the risk that more dramatic—and costly—interventions will be required. Pathogens like SARS-CoV and Ebola, which have limited presymptomatic transmission, have previously been contained using targeted measures like isolation of cases and quarantining of contacts. But this is more challenging for infections like influenza and SARS-CoV-2, for which substantial transmission can occur before cases become symptomatic [9]. Once human-to-human transmission establishes, such pathogens can be extremely difficult to control.

Many current front-line countermeasures for respiratory virus pandemics are medieval in origin, from quarantining travellers to shutting people in their homes. Although the G7 “100 Days Mission” aims to accelerate development of diagnostics, vaccines, and treatments for epidemic threats [10], it does not address the problem of how to respond in the meantime. Even if pharmaceutical tools were to become available within 100 days, countries would still have to face the threat without them for several months.

If human spillover and viral adaptation to efficient transmission happens rapidly, it may not be possible to intervene before substantial outbreaks. In this situation, countries must learn the lessons of the COVID-19 pandemic. How can we deploy targeted control measures that scale during an exponentially growing epidemic, limiting disruption to where transmission is happening rather than imposing blanket restrictions? How can we address the social inequities that drive risk in certain subgroups, enabling transmission chains to continue? What are the decision points for an effective early response amid uncertainty, and how do we avoid an ineffective wait-and-see approach when it comes to potential pandemics?

In hindsight, it may feel obvious what countries should have done in January 2020 in response to COVID-19. Although there were only a few dozen confirmed cases by mid-January, and mixed evidence around human-to-human transmission, it may seem now that the nature of the threat could only have gone in the direction of a pandemic. But we must overcome this hindsight and remember the situation and signals at the time. Because we will face such situations and signals again with future threats, whether H5N1 or something else. And a successful early pandemic intervention means effective action when outbreaks are small and outcomes uncertain.
